# Barriers and Challenges to Self-Care among Older Adults with Knee Osteoarthritis: A Qualitative Study

**DOI:** 10.4314/ejhs.v32i5.12

**Published:** 2022-09

**Authors:** Faranak Kooranian, Zohreh ParsaYekta, Maryam Rassouli

**Affiliations:** 1 Department of Nursing, PhD Candidate of Nursing and Midwifery, Tehran Medical Sciences, Islamic Azad University, Tehran, Iran; 2 Department of Nursing, Faculty of Nursing and Midwifery, Tehran Medical Sciences, Islamic Azad University, Tehran, Iran; 3 Cancer Research Center, Shahid Beheshti University of Medical Sciences, Tehran, Iran

**Keywords:** Osteoarthritis, Knee, Aged, Self Care, Qualitative Research

## Abstract

**Background:**

Knee osteoarthritis (KOA) is a chronic disease causing disability in older adults. Self-care is an effective strategy for KOA management in older adults and clinicians have shown increased interest regarding the challenges of self-care activities in these patients. The present study aimed to explore the perception of older adults' patients with KOA regarding barriers and challenges faced in their self-care management.

**Methods:**

In this qualitative study, data were collected using purposeful sampling and in-depth semi-structured interviews with 22 older adults with KOA, their first-degree caregivers, and medical team members in Mashhad, northeast Iran, from April to December 2020. All the interviews were transcribed verbatim. Interviews continued until reaching data saturation. All interviews were recorded. Data analysis was done using conventional content analysis according to the technique described by Graneheim and Lundman. The MAXQDA (Version 10) was used to organize, code, and manage the data.

**Findings:**

Three main themes (confusion, confrontation with increasing stresses, and social rupture) were emerged as the barriers and challenges to self-care among older adults with KOA.

**Conclusions:**

The results of this study provide a broad range of context-specific of barriers and challenges to self-care among older adults with KOA, which can be used for designing and implementing appropriate interventions to improve self-care in these patients.

## Introduction

Knee osteoarthritis (KOA) is a chronic disease and the main cause of physical disability among older adults. KOA can lead to joint impairment, followed by stiffness, edema, movement limitations, pain, and an increased risk of falling, which may affectan individual'sindependence and autonomy (1). KOA is now considered one of the ten most disabling diseases in developed countries causing limited mobility in 80% and with an additional 25% unable to perform activities of daily living (2). According to worldwide estimates, 250 million people are currently affected by OA of the knee(3). It is anticipated that the number of adults with arthritis increase to 78.4 million by 2040, most of whom will have KOA (4). KOA accounts for over 80% of the total OA disease burden, and its prevalence is rapidly increasing (5). In Iran, the rate of affliction and damages of KOA prevalence is 15.3% in the urban society and 19.3% in rural society, which is much higher than in other Asian countries (6). KOA has a strong association with aging(7), and it is estimated that 18% of women worldwide and 9.6% of men over 60 years of age suffer from KOA (2).

Low quality of life is one of the main consequences of KOA due to pain, social isolation, and poor psychological state (8). At present, there is no effective treatment for KOA (2–3). Therefore, self-care is an essential aspect of KOA management that comprises the actions and behaviors implemented by individuals towards monitoring and managing their chronic illness and maintaining health (9). Self-care programs of chronic diseases, particularly osteoarthritis (OA) among older adults are typically behavioral interventions that encourage older adults with KOA to play a significant role in managing their disease (10). Self-care is a multidimensional health-related concept, which includes activities that individuals do to keep their health and wellbeing (11). According to Orem's definition, selfcare behavior is a key concept in health promotion, which refers to the decisions made and activities done by a person to adapt to a health problem by improving his/her health (12). Limitations in mobility or self-care among older adults who suffer from KOA are associated with poor quality of life, future dysfunction, increased health and social care costs, and increased risk of mortality (7).

Because KOA assigns a considerable part of health care budgets to itself, reduction of costs and disease management development through disease-specific education for patients, targeted community-based programs, and self-care activities are considered as key goals (10). Self-care experiences are complex because individuals may have different viewpoints about their illness, so their priorities, expectations, and self-care methods may be different (9). Areason for a different aspect of self-care in every chronic disease is that people have a distinct conceptualization of their disease, its treatment, and their role in the treatment plan (13). Cultural and social differences can affect the understanding of the barriers and facilitators of self-care about diseases in each country (12). According to the qualitative literature review, barriers to self-care in chronic diseases may include reduced physical capacity, psychological problems, such as depression and anxiety, cognitive impairment, health literacy, improper self-care experiences, financial constraints including lack of health insurance coverage and the high cost of medications, lack of family/caregiver support, poor neighborhoods, and adverse environmental factors (14).

Identification of self-care barriers is a prerequisite to developing self-care interventions (15) providing the field for progression of self-care behaviors, keeping independence and autonomy, doing daily activities, and finally improving the quality of life of patients. Because it is necessary to identify barriers and challenges to self-care to design self-care improving interventions, the current study was conducted to determine barriers and challenges to self-care among older adults with KOA.

## Methods

In a qualitative study, which was conducted from April to December 2020, participants comprised of older adults with KOA referring to a Rheumatologists, the first-degree caregivers for older peoplewith KOA, and their medical teams in Mashhad, northeast Iran. Participants were selected based on the purposeful sampling method to match the aim of the research as closely as possible. Inclusion criteria of older adults with KOA were as follows: being able and willing to explain their experiences, being older than 65, having good mental health, ability to make verbal communication, and having at least 2-year experience of KOA. Caregivers were first-degree relatives of patients who were healthy physically and mentally. Inclusion criteria among medical team members were having at least 2-year experience of working on KOA patients. Sampling was determined using data saturation principles, with continual sampling until new data collected did not provide any new insights or themes on the phenomenon being studied. . The effort was to choose people with maximum variety of self-care activities, economic, social and educational backgrounds as well as widows, married, with and without children. The participants extensively talked about their problems; hence, the obtained data were diverse and rich.

**Data collecting**: The data were collected through in-depth, semi-structured, face-to-face interviews recorded as mp3 files. Participants were aware about recording of their voice. Two participants did not allow recording their voice, so the note-taking method was used. In total, 22 interviews were done: 14 interviews with older adults with KOA, 4 interviews with first-degree caregivers of patients with KOA, and 4 interviews with medical team members. Interviews were conducted in clinics, physiotherapy centers, patients' homes, local parks close to older adults' homes or nursing homes, upon request of participants and their convenience. Consistent with the objectives of the study, a semi-structured topic guide was developed based on an extensive review of the literature along with discussions with experts in the field. The following questions were asked: what are your experiences with this disease and caring for it? What self-care actions do you do? What are your problems in self-care? A question designed for first-degree caregivers and medical team: Do you have self-care experience of patients with KOA? What are the problems of patients in self-care behaviors and process? Data collection and analysis were simultaneous and during this process the topic guide was modified to explore emerging areas of interest. The duration of the interviews ranged from 34 to 45 minutes. Interviewswere conducted in a calm environment without the presence of others. All the interviews were conducted in Persian by the first author, a doctoral student trained in healthcare qualitative research.

**Data analysis**: All interviews were analyzed by usinga conventional content analysis method. As a systematic method, content analysis is used for analyzing verbal or written communication using a coding and categorizing approach. This method is suitable for exploring an individual's experiences and perception (16–17). In this study, the analysis utilized inductive content analysis, as described by Graneheim and Lundman (16). Data were analyzed in five steps. In the first step the interviews were read through and listened to several times by the first author to gain a sense of the whole. In the second step meaning units related to the aim were identified. In the third step the meaning units were condensed and labeled and finally coded on the basis of their content. Based on the codes, sub-categories and categories were developed in the fourth step. In the fifth step the categories were carefully discussed until main categories could be identified. All of the research team members validated the findings by reviewing and agreeing with the themes. The MAXQDA (Version 10) was used to organize, code, and manage the data.

**Rigor**: The criteria proposed by Lincoln and Cuba were used for establishing trustworthiness of the study findings using member checking, integrating the data sources and method integration, endorsing the coding by the colleagues familiar with qualitative research, coding, classifying similar codes and categories, transcribing the interviews as soon as possible and peer debriefing. In addition, the researcher carefully registered the research documentations to allow an external reviewer to evaluate the study.

**Ethical considerations**: The present study was approved by the ethics committee of Islamic Azad University of Medical Sciences under the ethics code of IR.IAU.TMU.REC.1399.170. The required permissions were obtained from Islamic Azad University ofMedical Sciences to introduce the researcher to research centers. Participants signed the consent form and informed their voluntary participation before the commencement of each interview. Emphasis was placed on the voluntary nature of the participants' involvement in the study. Participants were told that they could withdraw from the study at any time without giving a reason and theirnon-participation in the study did not interfere with their treatment and medical or nursing care process. Information confidentiality and privacy rules were observed. After consent letters (verbal and written) were signed, participants announced their satisfaction with the audio record, and they were ensured of information confidentiality, the data were gathered.

## Results

The Consolidated Criteria for Reporting Qualitative Research (COREQ) checklist was used to report important aspects of this study. Table 1 reports the characteristics of participants. Finally, 15 subcategories, 8 secondary categories, and 3 main categories (including confusion, confrontation with increasing stresses, and social rupture) were extracted from analyzed interviews (Table 2).

**Table 1:** Demographic characteristics of participants

Participants	Code	Gender	Age (year)	Marital status	Education degree	Disease duration (year)	Job	Interview time (min)
Older adults with KOA	1	Female	77	Deceased husband	Diploma	10	Housewife	45
	2	Female	67	Married	Diploma	12	Chef	40
	3	Female	68	Married	Diploma	10	Member of charity	42
	4	Female	81	Deceased husband	Secondary school	20	Housewife	35
	5	Female	72	Deceased husband	Elementary school	11	Housewife	38
	7	Male	67	Married	Diploma	5	Retired driver	36
	8	Female	66	Married	Illiterate	7	Housewife	35
	9	Female	65	Deceased husband	Associate degree of the operation room	35	Retired employee of the operation room	42
	11	Male	71	Married	BA	12	Retired accountant	45
	12	Female	65	Married	Elementary school	10	Housewife	39
	14	Female	73	Deceased husband	Illiterate	15	Housewife	35
	19	Female	79	Deceased husband	Illiterate	14	Housewife	34
	20	Female	65	Married	Secondary school	6	Housewife	37
	21	Male	78	Married	Diploma	8	Retired employee	36
First-degree caregivers	Code	Age (year)	Gender	Marital status	Education degree	Relationship with patient	Job	Interview time (min)
	6	Female	49	Married	Diploma	Daughter	Housewife	40
	10	Female	50	Married	BA in nursing	Daughter	Housewife	45
	13	Male	70	Married	Secondary school	Wife	Employed in a workshop	35
	22	Female	52	Single	BA in management	Daughter	Bank employee	42
Medical team	Code	Age	Gender	Marital status	Education	Work experience	Job	Interview time (min)
	15	Male	50	Married	Subspecialty	15	Rheumatologist (physician)	37
	16	Male	52	Married	Subspecialty	20	Rheumatologist	35
	17	Male	58	Married	MSc.	30	Physiotherapist	45
	18	Female	29	Single	BSc.	5	Physiotherapist	42

**Table 2:** Categories, secondary categories, and subcategories extracted from data

Main category	Secondary category	Subcategories
Confusion	Lack of awareness	Lack of knowledge about the disease, inability to use information sources
	Uncertainty	Fear of future, ambivalence, possible threats
Confrontation with increasing stresses	Disease stresses	Erosive process of disease, coping with aging diseases
	Care stresses	Financial difficulties, sense of loneliness
	Spiritual distress	The feeling of absurdity, sense of guilt
Social rupture	Role-playing disorder	Not being the authority, inability to make decisions
	Social isolation	Negative affections affecting the social relationships, despair

**Confusion**: According to participants' statements, the older people faces some issues, such as lack of awareness about the disease in form of lack of knowledge about the disease, inability to use information sources, and uncertainty with fear of future, ambivalence, and possible threats.

**Lack of awareness**: Having information and awareness about disease requires older adults ability to search through information sources to find the new treatment methods, achieve safe treatment, obtain new disease-related and irrelevant information, become aware of information sources for disease control, to obtain tips from reliable sources. This information helps the older adultswith KOA manage diseases and their symptoms and complications logically and make accurate self-care plans with emphasis on diseases knowledge. In this case, an 81-years old woman stated, “I am not literate so cannot use smartphone or internet to find a drug for my knee pain. I gave up!” “I do not do anything to mitigate my pain except for taking pain relievers and keeping my knee warm because do not know what to do” a 72-years old woman says.

**Uncertainty**: The unpredictability of disease complications causes fear of the future, ambivalence, and possible threats. Participants have described the fear of the future as fear of unsuccessful surgery or its consequences, fear of unknown, vague, and dark future, fear of absolute disability, and dependence on others. Hence, this fear leads to an unknown future in life. A 77-years old woman states, “I always think what I should do if I cannot walk anymore. I ask God not to make me dependent on my children. I prefer to die but not lose my autonomy. I am not sure about my future, and such thoughts make me stressed and disabled.” Moreover, decisions made on choosing the best treatment, accepting or rejecting surgery, as well as conflicting information about the disease and treatments make older people confused in terms of the best way for disease management. An 81-years old woman stated, “I talked to many people, some say go for an operation to relieve your pain, but some others warn me that surgery may be harmful. I am left with a dilemma. I have pain and fear at the same time, so I do not know what to do.”

In terms of possible threats, changes in feet appearance and walking disorder followed by KOA put the patient at risk of potential damage and affect the self-care causing dependence. In this case, a 72-years old woman stated, “I am careful not to fall because I know that I cannot get up if I fall.” “Patients may fall, and there is nobody to help them, so they may break their legs or hands,” added a 29-years old physiotherapist.

**Confrontation with increasing stresses**: The nature of old age causes many stresses for older adults. The occurrence of chronic diseases, such as KOA intensifies these challenges by creating stresses resulting from diseases, case stresses, and spiritual distress.

**Disease stresses**: The degenerative and progressive nature of the disease besides the elderlies' attempt for management of KOA and other chronic diseases occurring in old age cause erosive and chronic conditions. The erosive process of the disease means a gradual loss of energy and power of older adults leading to many psychological issues as KOA progresses. In this case, morale and motivation for life are reduced disturbing the living process. A 50-years old woman (patient's daughter) explained, “Unfortunately, this disease causes many pains and sufferings. I feel KOA makes stress, how a patient can tolerate such pain. The worse thing is problematic sitting down and standing up. The patient even cannot ascend or descend stairs, and this leads to immobility.”

Comorbidity during the age-old requires the ability to manage other diseases besides KOA. Otherwise, the older adults cannot make self-care plans, drugs are not taken timely, or they may forget to take medications, which cause side effects. A 78-years old man explained, “I suffer from high blood pressure and diabetes in addition to knee pain. I take 10 pills a day. I took boxes from the drugstore to write on them and separate drugs for the morning, noon, and night in order not to forget them. I will forget if I do not do that.”

**Care stresses**: Care stresses among older adults with KOA include financial difficulties and a sense of loneliness. Financial difficulties cause constraints on treatment and care costs, and concern for treatment and care persistence. The patient loses self-care ability due to lack of financial support and independence, and dependence on family. In this case, a 65-years old woman stated, “My sons want to pay the cost of surgery, but I cannot put my burden on the shoulders. I prefer to tolerate this pain.”

Sense of loneliness is another care stress that older adults face. Losing loved ones and a sense of loneliness during old age are inevitable experiences. The healthy older people can accept loneliness, but some chronic diseases, such as KOA cause movement limitations making older adults dependent on others. Therefore, such diseases prevent self-care by older people. In this case, a 77-years old woman stated, “When you are healthy but alone, you can do your tasks, you can go out, go shopping, swimming, etc. but I suffer from leg pain, so I cannot go out and stray home.”

**Spiritual distress**: Spiritual distress occurs when older people cannot do their religious tasks, so they feel dissatisfied. As older adults with KOA cannot manage and monitor home tasks and assign these routines, they feel concerned about the cleanness of the house. Moreover, older adults may feel absurd, indifferent, and guilty. Disability and limitations make older people change their religious procedures. Moreover, the vague goal of life, dissatisfaction, and worries affect self-care.

In case of a sense of guilt, a 77-years old woman explained, “I do not why this happened to me! I feel guilty and ask God what my sin was and why I should bear such pain as punishment.” “Sometimes, I feel guilty I think this is the punishment of my sin,” a 67-years old man added. A 73-years old woman explained, “I used to call God, but God did not hear my voice. I do not know what my sin is and have to tolerate such pain.”

**Social rupture**: The social rupture theme comprised factors related to the role-playing disorder and social isolation. In terms of role-playing disorder, not being an authority and inability to make decisions were introduced as important factors. Movement limitations followed by KOA in old age lead to role-playing disorder affecting the self-worth and capability of patients. Moreover, older adults feel less power in deciding on personal, familial, and social issues, feel absurd, and have less motivation and power to manage life. In this case, a 67-years old man stated, “I feel I am not productive anymore as if my opinions and ideas are not important for others, and this makes me weak and powerless.”

In terms of social isolation, negative affections affecting social relationships and despair were introduced as subcategories. Distancing from society, depression, isolation, disgust of life resulting from the disease, and lack of relationship with others make older adults powerless. A 67-yeard old mad explained, “My social behaviors have changed. I try to make fewer connections. I cannot even go for a walk or shopping. I have been isolated and distanced from society.” “I hate myself because I do not have mobility like past. Sometimes, this pain gets me bored of life,” added a 77-years old woman. Moreover, a sense of despair reduces motivation for self-care behaviors and attempts to have a better life. A participant stated, “I feel disappointed due to this pain and disability. I visited many doctors, but I could not find any effective treatment. I ask God to kill me to get rid of this pain!”

## Discussion

Knowledge and information about diseases and the ability to use information sources are prerequisites to self-care. Due to movement limitations and disability followed by KOA in older adults, these patients must obtain information about diseases and use information sources to cope with such limitations and disabilities. Some older adults with KOA who participated in this study named lack of knowledge and disability in using information sources as challenges and barriers to self-care. However, they explained that could not acquire new and effective information for disease self-management. Health literacy is defined as an individual's ability to process, analyze, and apply information to stay healthy. Strong evidence indicates that persons with lower levels of health literacy cannot understand the educational subjects, which this issue leads to nonadherence to self-management practices, such as following specific diets, taking medications intractably, and causing adverse health implications (14). If older people who suffer from KOA have lower health literacy to acquire knowledge and information, family caregivers with high literacy can help older adults deal with self-care challenges. However, studies show that some issues, such as lack of information, skill, and caregivers' poor perception of information are the main challenges in caring for patients(18). Indeed, patient information and education in evidence-based clinical guidelines are considered core treatments to allow patients to use this information to implement changes and make treatment decisions(19). The results of a qualitative studyonMalaysian older adults with KOA about the information required to enable them for self-managementrevealed that the patients' knowledge about KOA varied between individuals with many expressing that they needed more information about KOA (20). Also, another study showed that Japanese patients with KOA desired evidence-based information and to connect with other people in the same situation to solve problems related to their condition (21).

In the present study, some factors including fear of the future, ambivalence, and possible threats were introduced under the uncertainty that is one of the consequences of lack of awareness. Uncertainty in disease occurs when the patient cannot determine the meaning and evaluate disease-related events, and cannot predict disease consequences due to a lack of adequate symptoms; therefore, it is considered as a psychological stressor for the patient (22). Uncertainty affects a wide range of the patient's life, daily activities level, and quality of life (23). The older adults with KOA studied in this paper experienced fear and ambivalence due to implications resulting from diseases and mental issues, which affect decisions made on self-care, andare considered as a barrier to the self-care program implementation. Fear is an emotional reaction to a specific threat thatrefers to unknown fears caused by OA and its effect on daily and social life performance. Fear contributes to avoiding those painful or harmful activities, and this issueincreases disability through physical immobility and weakening the musculoskeletal system (24). Many participants felt dilemma and ambivalence in terms of making decisions on choosing the best treatment, accepting or rejecting surgery, and receiving conflicting information about the disease and its treatment, so they explained that this issue affected their self-care process. According to the studies conducted on the fear and ambivalence concerning surgical and pharmacological treatments, professionals should focus on the needs of the patients for alleviating pain, and delaying the disease progression (19). In the present study, some participants pointed to possible threats, falling risk, or further damages. In patients with KOA, falls may cause fractures, tissue injuries, and even death, and fear of falling occurs with gait changes and disorders, reduction in confidence, depression, feelings of inadequacyin performing daily life activities, and losing active lifestyle. KOA restricts an individual physically, psychologically, and socially, so individuals face problems in some functions, such as getting up from a seat, and descending stairs (25). In the presence of such barriers, the individual loses motivation to perform self-care programs.

In terms of disease stresses caused by the disease, some factors such as the erosive process of disease, and coping with aging diseases were introduced. Older adults with KOA in this study explained that they lost their power and energy for self-care as diseases progressed and symptoms intensified, and this issue led to some changes in their normal life. In a qualitative study, the diversity in the experienced symptoms of KOA, such as knee pain, stiffness, difficulties in daily activities, reduction of work, instability, weakness, lack of mobility, and psychological impactaffected the whole body and different life levels of patients(19). Moreover, OA comorbidity with aging may have synergistic effects on mobility and self-care limitations. Hence, identifying these synergistic effects is important to clinical practice. The most prevalent comorbidities among people with hip/knee OA include cardiovascular diseases, depression, type 2 diabetes, hypertension, and other types of musculoskeletal pain (7). It seems that management of these diseases besides knee OA is the main challenge for self-care. In terms of care stresses, financial difficulties, and a sense of loneliness were extracted. In terms of financial difficulties, in the present study, some older people with KOA explained that their independence and autonomy were reduced, so their dependence on the family was increased due to lack of financial support and inability to afford care and treatment costs. KOA is a major public health problem and incurs many indirect and direct healthcare costs (26). Moreover, it will have considerable social and economic consequences (5). In addition, the results of the present study indicated that a sense of loneliness reduced the motivation and desire of a person for self-care; hence, it is considered as a barrier to self-care. Loneliness during old age is an unpleasant, negative, and painful experience that causes some feelings, such as boredom, inefficiency, despair, depression, and anxiety (27). Social isolation and loneliness have detrimental effects on older people's physical and mental health (28). Loneliness experiencedduring aging after losing loved ones is an inevitable phenomenon. Healthy older adults can accept loneliness, but this feeling occurs with disabling diseases, such as knee OA. Tolerating pain and disability caused by diseases and a sense of loneliness have considerable effects on the mental health of an individual and reduce an individual's self-care motivation. Moreover, if lack of financial support and loneliness occur simultaneously then the tension of individuals will be intensified affecting their independence in doing self-care practices. In the current study, feeling of absurdity, and a sense of guilt was introduced as subcategories of spiritual distress. Because human biology is composed of physical, mental, social, and spiritual dimensions, human health also depends on the health of these dimensions; hence, behaviors and abilities of humans are originated from the performance of and interaction between these four dimensions. Spiritual wellbeing is one of the important health dimensions connecting inner forces.

In the present study, conflicts between religious beliefs in addition to a sense of absurdity, and guilt reduced motivation for doing self-care measures. The religious dimension of life is an important source of strength, and coping for many people, contributes to welfare and security, is beneficial for mental and physical health, increases life satisfaction, and prevents substance abuse, suicide, and depression (29). Spiritual and religious activities are self-care behaviors and are described as activities providing strength and emotional support (30). A negative perception of aging causes a sense of guilt even in theolder adults, who do not have physical and psychological restrictions(31). If these negative perceptions occur with disabling diseases, such as KOA, they will affect welfare, security, adaptation, life satisfaction, and subsequently selfcare motivation. Older adults with KOAfeel guilty if they do not follow the diet advice and do not meet the expectations of the people closest to them (19).

Older adults try to participate in decisions made on self-care, be independent, and keep their autonomy (32). Negative affections affecting social relationships and despair were extracted as subcategories of social isolation. Results of studies showed that communication with others and feelings of support are important self-care predictors (33). Distancing from community and depression feeling, isolation, and life hatred due to illness, and lack of communication with others cause losing morale and ability to do things. Previous studies suggested there is an association between musculoskeletal pain and social isolation. The signs and symptoms commonly associated with OA, especially joint pain and reduced function may increase the risk of social isolation. Since social isolation is a potentially reversible condition, it has been attempted to identify social isolation in this group of patients in the early stages (34). Some participants explained they were reluctant to do their daily activities and self-care practices due to despair and lack of motivation. Hope is always good and desirable and serves as an individual, mental, and cognitive perceived capability for identifying routes to desires and creating motivation to achieve positive goals and avoid negative consequences. Hope is a synonym for some positive concepts, including optimism, self-esteem, wellbeing, and happiness (35). Lack of mobility caused by knee OA may cause loss of independence. Loss of independence leads to social isolation because the patient cannot enter the communityas they could enter in the past. This isolation from the community causes mental distress and despair (36). There are some limitations in the present study that need to be addressed. A limitation of current study may be recall bias since participants addressed their current experience and sometimes previous experiences also. The lack of generalizability of the findings to all older adults' patients with KOA in different geo-cultural contexts, due to small sample size and participants' socio-demographic and cultural characteristics, are another limitations of this study.

In conclusion, the results of this study provide a broad range of context-specific of barriers and challenges to self-care among older adults with KOA, which can be used for designing and implementing appropriate interventions to improve self-care in these patients.
